# Volumetric Analysis of Thalamic Nuclei in Epilepsy Patients: A Sex-Based Comparative Study

**DOI:** 10.3390/diagnostics16131999

**Published:** 2026-06-26

**Authors:** Anıl Kaya, Turgay Karataş, İpek Balikci Cicek, Mehmet Akçiçek, Merve Aydın, Hıdır Pekmez

**Affiliations:** 1 Department of Anatomy, Faculty of Medicine, Malatya Turgut Özal University, 44210 Malatya, Türkiye; merve.aydin@ozal.edu.tr (M.A.); hidir.pekmez@ozal.edu.tr (H.P.); 2 Department of Anatomy, Institute of Health Sciences, İnönü University, 44280 Malatya, Türkiye; 3 Department of Anatomy, Faculty of Medicine, İnönü University, 44280 Malatya, Türkiye; turgay.karatas@inonu.edu.tr; 4 Department of Biostatistics and Medical Informatics, Faculty of Medicine, İnönü University, 44280 Malatya, Türkiye; ipek.balikci@inonu.edu.tr; 5 Department of Radiology, Faculty of Medicine, Malatya Turgut Özal University, 44210 Malatya, Türkiye; mehmet.akcicek@ozal.edu.tr

**Keywords:** epilepsy, thalamus, volbrain, MRI, sex differences

## Abstract

**Background/Objectives**: Epilepsy is a long-term condition marked by recurring seizures that greatly affect daily life. The thalamus, with its extensive connections to the cortex, plays a vital role in epileptic processes. Changes in the volume of the thalamic nuclei are believed to occur in epilepsy. Our study aimed to examine the volumetric characteristics of thalamic nuclei in individuals diagnosed with generalized epilepsy and to explore the potential role of changes in these nuclei in the process of epileptogenesis. **Methods**: In this retrospective study, a total of 232 participants were included in the study. 1.5T MRI images of epilepsy patients and age- and sex-matched healthy individuals were analyzed. Thalamus nucleus volumes were measured using the VolBrain automatic segmentation system. Right–left volumetric comparisons of homologous thalamic nuclei were performed within each study group to evaluate hemispheric lateralization patterns. Groups were compared based on sex. FDR correction and ANCOVA adjustment for ICV and age were applied; significance threshold was *p* < 0.05 after correction. **Results**: After applying false discovery rate (FDR) correction, significant differences were observed in multiple thalamic nuclei in the epilepsy group compared to controls. In women, volume increases were confirmed in the right VAN, bilateral VPLN, and left MGN, while bilateral MTN volumes were significantly reduced and right ISN volume was lower in the epilepsy group. In men, only bilateral MTN volumes remained significant after FDR correction; no nucleus survived after additional ANCOVA adjustment for intracranial volume and age. Right–left comparisons revealed consistent lateralization patterns, with AVN showing right-sided predominance and VAN showing left-sided predominance across both sexes after FDR correction. **Conclusions**: After correction for multiple comparisons and adjustment for intracranial volume and age, only a limited number of thalamic nuclei remained significantly altered, predominantly in female patients. These findings suggest that thalamic involvement in generalized epilepsy may be selective rather than widespread and may vary according to sex.

## 1. Introduction

Epilepsy is a non-contagious neurological disorder characterized by epileptic seizures caused by abnormally high electrical activity in cortical neurons [1]. Epileptic seizures occur abruptly due to transient abnormal neuronal activity [2]. Studies have shown that recurrent seizures can cause both functional and structural changes. Morphological alterations, such as volume loss and synaptic reorganization, have been observed in specific brain regions, especially in individuals with a long history of epilepsy. With advances in neuroimaging techniques, structural abnormalities in certain brain regions have been identified in approximately 21–37% of patients with epilepsy [3]. These regions include the hippocampus, amygdala, cerebral cortex and basal ganglia, as well as deep brain structures such as the thalamus [4].

Various studies in the literature indicate that changes in thalamus volumes may also occur in patients with epilepsy [5,6,7]. The thalamus is a vital structure in seizure development. However, the underlying mechanisms of seizures can differ among various epileptic syndromes [8]. Structural and functional abnormalities in the corticothalamic pathways are believed to play a crucial role in the pathophysiology of seizures [9].

Understanding the mechanisms of epilepsy-related seizures involves not only examining volumetric changes in the thalamus but also exploring the functional roles of specific nuclei groups in this process. The anterior, pulvinar, and mediodorsal nuclei, in particular, have been shown to play significant roles in the cortical correlates of epileptic activity [10,11].

Epileptic seizures are believed to originate not only from issues in isolated cortical areas but also from imbalances across multiple neural networks spanning different brain regions. While reciprocal connections between the thalamus and cortex within these networks typically maintain cognitive function and sensory regulation, disruptions in these interactions during epileptic activity can promote seizure spread. Specifically, synchronization problems in thalamocortical pathways can lead to the buildup of electrical bursts and the appearance of clinical symptoms. Functional disturbances in this system may influence both seizure initiation and subsequent processes [12,13,14].

Today, advanced neuroimaging techniques enable detailed examination of microstructural and volumetric changes in the brains of patients with epilepsy. Magnetic resonance imaging (MRI) and voxel-based morphometry (VBM) provide quantitative assessments of nuclear changes in the thalamus. These technologies are valuable tools not only for understanding the disease’s pathophysiology but also for monitoring disease progression and evaluating treatment responses [15,16].

The thalamus is a key target in epilepsy treatment, especially for seizure control. Neuromodulation techniques like deep brain stimulation (DBS) specifically focus on the anterior and intralaminar nuclei of the thalamus. These methods aim to lower seizure frequency and severity. Such approaches support the idea that the thalamus plays a regulatory role in epileptic processes and is central to seizure spread. Therefore, understanding how structural changes in thalamic nuclei affect epileptogenesis is essential for developing effective treatment strategies [17,18].

It is often noted in the literature that changes in brain structure and function in epilepsy may vary across different subtypes of the condition [19]. In the present study, only patients with generalized epilepsy were included, allowing for the evaluation of thalamic nuclei within a more homogeneous clinical group. Generalized epilepsy is characterized by the widespread involvement of thalamocortical networks, which are known to play a central role in seizure generation and propagation. These common pathophysiological mechanisms may lead to distinct structural and functional alterations in brain tissue, particularly within thalamic nuclei. Therefore, this study focuses on the structural and functional effects of generalized epilepsy and highlights the importance of thalamic alterations in this subtype. In this context, our study aimed to examine the volumetric characteristics of thalamic nuclei in individuals diagnosed with generalized epilepsy and to explore the potential role of changes in these nuclei in the process of epileptogenesis.

## 2. Materials and Methods

### 2.1. Study Design and Participants

This retrospective study was conducted in collaboration with the Departments of Neurology and Radiology at Malatya Training and Research Hospital. Hospital records were thoroughly searched between January 2019 and December 2022. Ethical approval was obtained from the Malatya Turgut Özal University Clinical Research Ethics Committee (approval date: 6 May 2025, decision no: 2025/108). The research adhered to the principles of the Declaration of Helsinki.

A total of 232 individuals participated in the study; the study groups included 58 female patients with epilepsy, 58 male patients with epilepsy, 58 healthy male controls, and 58 healthy female controls. The study findings reported throughout the manuscript are based on the full dataset comprising 232 participants. The epilepsy group was diagnosed based on clinical evaluation, seizure semiology, electroencephalography (EEG) findings, and neurologist assessment. Only patients diagnosed with generalized epilepsy were included in the study. The mean epilepsy duration was approximately 3.7 years (range: 3–11 years). The control group comprised individuals who underwent MRI for non-specific complaints such as headache or dizziness and were reported to be neurologically healthy with normal brain structures.

All participants’ thalamic nuclei were measured using automated segmentation, including the Anterior Ventral Nuclei (AVN), Lateral Geniculate Nuclei (LGN), Ventral Anterior Nuclei (VAN), Ventral Lateral Anterior Nuclei (VLAN), Ventral Lateral Posterior Nuclei (VLPN), Ventral Posterior Lateral Nuclei (VPLN), Pulvinar Nuclei (PN), Medial Geniculate Nuclei (MGN), Centromedian Nuclei (CN), Mediodorsal Nuclei (MN), Habenular Nuclei (HN), Mammillothalamic Tract Nuclei (MTN), ISN (Intermediate Space Nuclei). This comprehensive assessment allowed for detailed volumetric analysis and right–left comparisons across all major thalamic subregions.

### 2.2. Inclusion and Exclusion Criteria

Inclusion criteria for the epilepsy group included a confirmed diagnosis of epilepsy by a neurologist, availability of T1-weighted MRI images, and age between 18 and 65. For both groups, individuals with a history of head trauma or previous cranial surgery, other neurological or psychiatric conditions, systemic diseases that could impact brain morphology, or poor-quality or incomplete MRI data, patients with visible structural brain lesions on MRI (such as tumors, stroke, or major structural abnormalities) were excluded from the study.

### 2.3. MRI Scan

All MRI examinations were conducted on a SIEMENS Amira (Erlangen, Germany) 1.5 Tesla scanner. Structural images were captured using a 3D T1-weighted magnetization-prepared rapid gradient-echo (MPRAGE) sequence. The parameters included repetition time (TR) = 2200 ms; echo time (TE) = 2.79 ms; flip angle = 8°; field of view (FOV) = 250 mm; slice thickness = 1.0 mm; number of slices = 192; matrix size = 256 × 256.

### 2.4. Image Processing and Volumetric Analysis

Volumetric analyses were conducted using the VolBrain platform (https://volbrain.net/home; accessed on 31 July 2025). a web-based, fully automated system for measuring brain volume. This method reduces human interaction and thus minimizes interobserver variability. All volumetric values were obtained from the VolBrain platform and are reported in cubic centimeters (cm^3^). Because nuclear volumes are small, their values appear as decimal fractions of cm^3^. In our study, measurements focused specifically on the thalamus and its main nuclei. All segmentations were visually inspected for obvious segmentation errors before inclusion in the final analysis. All data collected were used for statistical analysis. The VolBrain platform has previously demonstrated good reliability and accuracy for automated brain structure segmentation and volumetric assessment in MRI studies and has been validated against expert manual segmentation methods [9,20].

### 2.5. Statistical Analysis

Qualitative data from the variables included in the study were summarized as numbers (percentages), and quantitative data were summarized as medians (minimum–maximum) or mean ± standard deviation. The distributional characteristics of quantitative data were assessed using the Kolmogorov–Smirnov test. In comparisons of two independent groups, the independent samples *t*-test or the Mann–Whitney U test was used where appropriate, according to a normal distribution. In pairwise right–left comparisons of thalamic nucleus volumes, the paired-samples *t*-test was used for normally distributed data and the Wilcoxon signed-rank test for data that did not conform to normal distribution; normality was assessed separately for right and left values of each nucleus. To correct for multiple comparisons, the Benjamini–Hochberg false discovery rate (FDR) procedure was applied separately for between-group comparisons (28 tests per sex) and right–left paired comparisons (14 tests per group). Analysis of covariance (ANCOVA) was additionally performed for all between-group comparisons with intracranial volume (ICV) and age entered as covariates, followed by FDR correction of the resulting *p*-values. ANCOVA assumptions were evaluated for all models in both sexes. Homogeneity of regression slopes was tested by including group × covariate interaction terms (group × age, group × ICV); a significant interaction was interpreted as a violation of this assumption. Residual normality was assessed using the Shapiro–Wilk test applied to model residuals. For the nuclei surviving ANCOVA-FDR correction in female participants, the homogeneity of regression slopes assumption was satisfied in all cases, and residual normality was satisfied for the majority of these nuclei. In male participants, no nucleus survived ANCOVA-FDR correction, indicating that the unadjusted findings in this group require confirmation in future studies. A *p*-value of <0.05 was accepted as the threshold for significance in all analyses. Effect sizes were calculated for all comparisons: Cohen’s d with 95% confidence intervals was reported for parametric tests, and rank-biserial correlation r was reported for non-parametric tests. Effect size magnitudes were interpreted as small (d = 0.2–0.5; r = 0.1–0.3), medium (d = 0.5–0.8; r = 0.3–0.5), and large (d > 0.8; r > 0.5) according to conventional thresholds. Partial eta-squared (η^2^p) was calculated as a measure of effect size for the ANCOVA group effect, with magnitudes interpreted as small (η^2^p = 0.01–0.06), medium (η^2^p = 0.06–0.14), and large (η^2^p > 0.14) according to conventional thresholds. Descriptive statistics and normality tests were conducted using IBM SPSS Statistics 26.0 for Windows (New York, NY, USA); FDR correction and ANCOVA were performed using Python 3 (scipy, statsmodels, pandas libraries).

Hemispheric asymmetry was assessed by directly comparing the volumes of homologous right and left thalamic nuclei within each study group. Paired statistical tests were used according to data distribution characteristics.

## 3. Results

In female participants, age and total intracranial volume values were similar between the epilepsy and control groups, and no significant differences were found in overall thalamic volumes. In nuclear comparisons, after applying false discovery rate (FDR) correction across all 28 nucleus comparisons (Benjamini–Hochberg procedure), significant volume increases were observed in the right VAN (*p* = 0.011, pFDR = 0.043, d = 0.482 [0.113, 0.852]), bilateral VPLN (right: *p* = 0.005, pFDR = 0.032, d = 0.537 [0.167, 0.908]; left: *p* < 0.001, pFDR = 0.003, d = 0.681 [0.306, 1.055]), and left MGN (*p* = 0.007, pFDR = 0.032, d = 0.512 [0.142, 0.882]) in the epilepsy group compared to controls. In the control group, bilateral MTN volumes were significantly higher than in the epilepsy group (right MTN: *p* < 0.001, pFDR < 0.001, r = 0.490; left MTN: *p* < 0.001, pFDR < 0.001, r = 0.475), and right ISN volume was significantly reduced in the epilepsy group (*p* = 0.007, pFDR = 0.032, r = 0.292). Left VAN (*p* = 0.039, pFDR = 0.110), right VLPN (*p* = 0.035, pFDR = 0.108), and left ISN (*p* = 0.024, pFDR = 0.082) did not survive FDR correction and are not considered statistically significant findings.

After ANCOVA adjustment for intracranial volume and age, right VAN (ANCOVA-pFDR = 0.041, η^2^p = 0.062, medium), bilateral VPLN (right: 0.025, η^2^p = 0.077, medium; left: 0.001, η^2^p = 0.128, medium), left MGN (0.041, η^2^p = 0.066, medium), and left MTN (0.001, η^2^p = 0.128, medium) remained significant. Right MTN, despite surviving FDR correction, did not remain significant after ICV and age adjustment (ANCOVA-pFDR = 0.396), suggesting that this difference may be partially confounded by these variables. Detailed comparisons are presented in Table 1.

In right–left comparisons within the female epilepsy group, AVN showed robust right-side dominance (AVN right: 0.168 ± 0.052 cm^3^ vs. left: 0.067 ± 0.032 cm^3^; *p* < 0.001, pFDR < 0.001, d = 1.613 [1.223, 2.004]) and VAN showed robust left-side dominance (VAN left: 0.168 ± 0.048 cm^3^ vs. right: 0.060 ± 0.023 cm^3^; *p* < 0.001, pFDR < 0.001, d = −2.274 [−2.761, −1.786]), both surviving FDR correction. Left MGN was significantly larger than right MGN in the epilepsy group (*p* = 0.009, pFDR = 0.040, d = −0.358 [−0.623, −0.092]). In the control group, AVN right-side dominance (*p* < 0.001, pFDR < 0.001, r = 0.999), VAN left-side dominance (*p* < 0.001, pFDR < 0.001, d = −2.026 [−2.476, −1.577]), and right MN dominance (*p* < 0.001, pFDR = 0.002, d = 0.497 [0.224, 0.770]) survived FDR correction. The previously reported right-side PN dominance in female controls (*p* = 0.040) did not survive FDR correction (pFDR = 0.140) and is not considered a significant finding. Right–left comparisons are presented in Table 2.

In male participants, no significant differences were observed between the epilepsy and control groups in age, intracranial volume, or total thalamic volume. After FDR correction, only bilateral MTN volumes showed statistically significant differences: both right MTN (*p* < 0.001, pFDR = 0.006, r = 0.399) and left MTN (*p* = 0.002, pFDR = 0.031, r = 0.330) were significantly reduced in the epilepsy group compared to controls. Left AVN (*p* = 0.016, pFDR = 0.146), left VAN (*p* = 0.029, pFDR = 0.172), and left VPLN (*p* = 0.031, pFDR = 0.172), which appeared significant in the uncorrected analysis, did not survive FDR correction and are therefore not reported as significant findings. After additional ANCOVA adjustment for ICV and age, bilateral MTN also lost significance (ANCOVA-pFDR = 0.291 for both), indicating that the group differences observed in male participants are substantially accounted for by variation in ICV and age. Detailed comparisons are presented in Table 3.

In right–left comparisons within the male epilepsy group, AVN right-side dominance (AVN right: 0.180 ± 0.055 cm^3^ vs. left: 0.081 ± 0.030 cm^3^; *p* < 0.001, pFDR < 0.001, d = 1.886 [1.457, 2.315]) and VAN left-side dominance (VAN left: 0.175 ± 0.053 cm^3^ vs. right: 0.069 ± 0.026 cm^3^; *p* < 0.001, pFDR < 0.001, d = −2.098 [−2.559, −1.638]) were confirmed after FDR correction. Right MN dominance also survived FDR correction in the male epilepsy group (*p* = 0.003, pFDR = 0.015, d = 0.404 [0.137, 0.672]). The previously reported left PN dominance (*p* = 0.022) did not survive FDR correction (pFDR = 0.078) and is not considered a significant finding. In the male control group, AVN right-side dominance (*p* < 0.001, pFDR < 0.001, d = 2.024 [1.575, 2.474]) and VAN left-side dominance (*p* < 0.001, pFDR < 0.001, d = −1.788 [−2.203, −1.373]) were similarly robust. Right MGN dominance (*p* = 0.040) and right MN dominance (*p* = 0.045) in male controls did not survive FDR correction (pFDR = 0.144 for both). Right–left comparisons are presented in Table 4.

**Figure 1:**
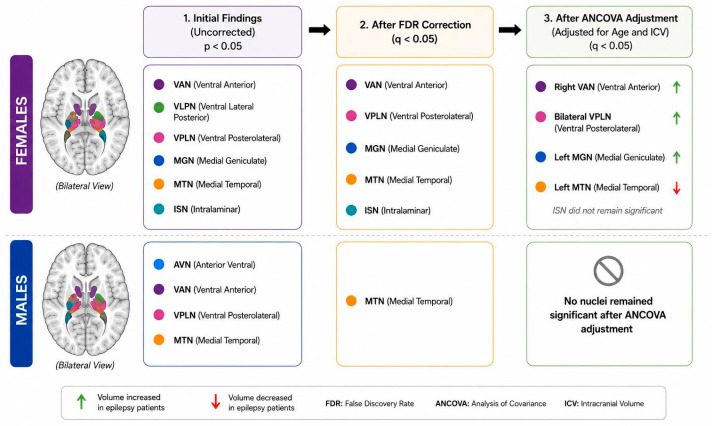
Workflow of statistical refinement and final significant thalamic nuclei in epilepsy patients compared with healthy controls. Green arrows indicate increased volumes in epilepsy patients relative to controls, whereas red arrows indicate decreased volumes.

Figure 1 summarizes the progression of statistically significant thalamic nuclei identified in sex-stratified analyses.

**Figure 2:**
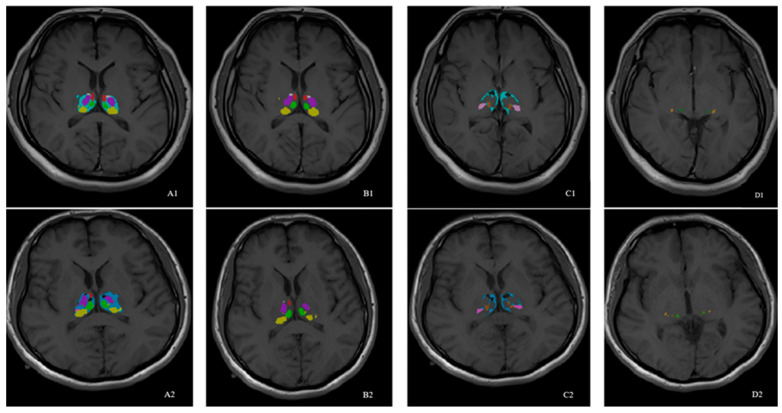
Representative axial MRI slices showing thalamic nuclei segmentations. (**A1**,**B1**,**C1**,**D1**) An epilepsy patient; (**A2**,**B2**,**C2**,**D2**) a healthy control. (**A1**,**A2**) General view of the thalamic nuclei. (**B1**,**B2**) Red: Anterior Ventral Nucleus; White: Ventral Lateral Anterior Nucleus; Purple: Ventral Lateral Posterior Nucleus; Yellow: Pulvinar Nucleus; Light green: Mediodorsal Nucleus. (**C1**,**C2**) Turquoise: Intermediate Space Nucleus; Black: Ventral Anterior Nucleus; Pink: Ventral Posterior Lateral Nucleus; Brown: Centromedian Nucleus. (**D1**,**D2**) Orange: Lateral Geniculate Nucleus; Green: Medial Geniculate Nucleus. The segmentation was performed using the VolBrain platform.

Figure 2 presents representative segmentation results. Axial MRI slices from an epilepsy patient and a healthy control demonstrate the thalamic nuclei automatically identified using the VolBrain platform. The color-coded regions correspond to the anterior, ventral, pulvinar, mediodorsal, geniculate, and centromedian nuclei, illustrating the accuracy and distribution of the segmentation.

## 4. Discussion

This study aimed to compare the structural characteristics and right–left volumetric patterns of the thalamus in patients with epilepsy and healthy controls.

The findings indicate alterations in a limited number of thalamic nuclei, particularly in female participants after correction for multiple comparisons and adjustment for covariates. These findings may indicate selective rather than widespread thalamic involvement in generalized epilepsy. However, because significance was retained only in a small subset of thalamic nuclei after statistical correction, the results should be interpreted with caution. The right–left differences observed in both groups suggest the presence of physiological hemispheric lateralization within several thalamic nuclei. However, the functional significance of these volumetric patterns remains uncertain. These findings suggest an association between epilepsy and alterations in thalamic nuclei volumes; however, it remains unclear whether these changes represent a cause or a consequence of the disease.

### 4.1. Right–Left VAN (Ventral Anterior Nucleus)

In female patients, right VAN volume was significantly larger in the epilepsy group compared with controls after FDR correction (p~FDR~ = 0.043), a finding further supported by ANCOVA adjustment for ICV and age (ANCOVA-p~FDR~ = 0.041). Left VAN showed a nominally significant difference (*p* = 0.039) that did not survive FDR correction (p~FDR~ = 0.110) and is therefore not considered a statistically significant finding. This enlargement may reflect structural alterations in thalamic networks involved in motor planning [21]. Normally, this nucleus transmits information to the premotor cortex by balancing inhibitory signals from the pallidum with excitatory signals from the cerebellum [22]. In epilepsy, when this balance is disrupted, the load on the thalamus may increase. Over time, this can lead to structural changes in neurons, gliosis, and strengthened synaptic connections, resulting in increased VAN volumes. Although left VAN volume appeared increased in male patients in the uncorrected analysis, this finding did not survive FDR correction and subsequent adjustment for age and intracranial volume and was therefore not considered a statistically robust finding.

### 4.2. Right–Left VPLN (Ventral Posterolateral Nucleus)

An increase in VPLN volume was observed in the female epilepsy group. Given the established role of the VPLN in somatosensory information processing and transmission, these findings may reflect altered somatosensory processing and thalamocortical network organization in epilepsy. Previous studies have suggested that recurrent epileptic activity may induce structural remodeling within thalamic nuclei involved in sensory integration [23,24]. Previous studies have suggested that chronic alterations within the VPLN may be associated with changes in somatosensory processing. These findings suggest that sensory disturbances accompanying epilepsy may be associated with thalamus-based neuroplastic changes [25,26,27].

In our study, the ventral posterolateral nucleus (VPLN) was found to be significantly larger in female epilepsy patients, suggesting that its role in integrating sensory transmission and motor control may be subjected to excessive load under epileptic activity. Although females generally show more widespread interhemispheric connectivity, as reported in previous structural connectome studies, whether this directly influences thalamic volumetric changes in epilepsy remains uncertain. However, this anatomical characteristic may partly contribute to the bilateral tendencies observed in our female group [28]. This may have resulted in a more generalized “bilateral load” manifesting as volume increase in females. In male epilepsy patients, left VPLN showed a nominally significant difference in the uncorrected analysis (*p* = 0.031); however, this did not survive FDR correction (pFDR = 0.172) and is therefore not considered a confirmed finding.

In contrast, another study did not report a significant volume difference in the VPLN [29].

This discrepancy may result from differences in clinical characteristics of the patient groups (seizure frequency, age at onset) or from variations in segmentation and volumetric methods, while our findings suggest that the VPLN may be involved in thalamocortical sensory networks affected in epilepsy; however, the clinical implications of this observation remain uncertain, and the absence of changes in this nucleus in the other study highlights the heterogeneous structure specific to epilepsy subgroups and indicates that thalamic networks may be affected at different levels. The VPLN increase may reflect a neuroplastic adaptation arising from repeated somatosensory input.

### 4.3. Left MGN (Medial Geniculate Nucleus)

In female epilepsy patients, the left MGN was found to be significantly larger compared to controls, suggesting that its role in auditory information transmission may be affected by epileptic networks, since the MGN is located along the main auditory pathway to the temporal cortex. This may reflect alterations in auditory thalamocortical processing [30]. Although a similar trend was observed on the right side, the lack of significance suggests that the change may exhibit a lateralized characteristic.

Similar studies in the literature support this finding; for example, Lee et al. (2023) reported a significant increase in the right medial geniculate nucleus (MGN) volume in patients with occipital lobe epilepsy (OLE) compared to healthy individuals [31]. This finding suggests that epileptic networks may affect auditory information transmission and that the MGN may play a role in this process; the absence of significant asymmetry in the control group indicates that this may represent a lateralized adaptation specific to epilepsy. No significant MGN alterations were observed in male epilepsy patients. Therefore, the volumetric changes identified in females may represent a sex-specific structural pattern; however, the functional implications of this finding remain unclear.

### 4.4. Right–Left MTN (Mamillothalamic Tract Nuclei)

Bilateral MTN reductions were observed after FDR correction in both female and male epilepsy patients. However, after adjustment for age and intracranial volume, only left MTN remained significant in females, whereas the findings in males no longer reached statistical significance. Since the MTN plays a central role in limbic and motor thalamocortical circuits, combined neuronal energy stress and oxidative stress may lead to neuronal loss and atrophy. Additionally, secondary degenerative processes such as epilepsy-related gliosis and microglial activation may reinforce the volume reduction. Long-term antiepileptic drug use and neurodevelopmental or genetic factors may also contribute to this thalamic volume decrease [23,24,25]. Considering the known involvement of the mammillothalamic tract in cognitive and motor circuits, the observed volume reduction may warrant further investigation. However, no conclusions regarding its functional consequences can be drawn from the present data [29,31,32]. This discrepancy may stem from the anatomical structural heterogeneity of the MTN and the variability of neuronal density across regions. Additionally, MTN may be less directly affected by epileptic activity compared to ventral or lateral nuclei, which could explain why some studies failed to detect volume changes statistically.

### 4.5. Right–Left MN (Mediodorsal Nucleus)

In male epilepsy patients, the right MN was observed to be significantly larger than the left, suggesting that limbic connections and emotional processes may be laterally affected in epilepsy. In females, no significant differences were observed either in comparison with controls or between the right and left sides. However, a natural asymmetry between the right and left MN is evident in the female control group. This finding may indicate that physiologically existing hemispheric differences in females become attenuated during epileptic processes, resulting in a more symmetrical distribution. Thus, while epilepsy appears to reinforce existing lateralization in males, it seems to balance natural asymmetries in females. These distinct patterns highlight that sex-specific neuroplasticity mechanisms may play a determining role in the effects of epilepsy on thalamic nuclei.

### 4.6. Right–Left ISN (Intermediate Space Nuclei)

In our study, a significant volume reduction was observed specifically in the right ISN in female epilepsy patients compared to the control group after FDR correction (pFDR = 0.032); however, this difference did not survive ANCOVA adjustment for ICV and age (ANCOVA-pFDR = 0.051), suggesting a possible confounding influence. Left ISN showed a nominally significant reduction (*p* = 0.024) that did not survive FDR correction (pFDR = 0.082) and is not reported as a significant finding. No significant differences were found between the right and left hemispheres within the female epilepsy group. In males, no significant changes in ISN volume were detected in comparison with controls or between hemispheres. Although the observed ISN alterations did not remain significant after comprehensive statistical adjustment, the nucleus remains of interest due to its role in attention, arousal, and pain modulation within thalamocortical networks [33]. Further studies are needed to clarify its potential involvement in generalized epilepsy.

### 4.7. Sex-Specific Findings in Male Patients

One of the most notable findings of the present study was the difference in thalamic involvement between female and male patients with generalized epilepsy. Although several thalamic nuclei appeared to differ from controls in the initial analyses, only a limited number of alterations remained robust after controlling for potential confounding factors. Interestingly, significant volumetric abnormalities persisted predominantly in female patients, whereas no nucleus remained significantly different in males after adjustment for age and intracranial volume. Partial eta-squared values for these adjusted findings in females ranged from 0.062 to 0.128, corresponding to medium effect sizes according to conventional thresholds.

These findings may indicate that the structural consequences of generalized epilepsy are not distributed uniformly across sexes. Previous studies have suggested that sex hormones, differences in brain maturation, and distinct patterns of network organization may influence the susceptibility of specific neural circuits to epileptic activity. The persistence of alterations in nuclei involved in sensory integration, thalamocortical communication, and limbic connectivity in female patients may therefore reflect a greater vulnerability of these networks to the long-term effects of epilepsy.

The disappearance of several initially observed findings after comprehensive adjustment suggests that demographic and anatomical factors may contribute to some of the volumetric differences reported in epilepsy studies and should therefore be carefully considered when interpreting structural alterations. Consequently, the identification of epilepsy-related structural biomarkers requires careful consideration of both biological covariates and sex-specific effects.

Taken together, our findings support the concept that generalized epilepsy is associated with selective rather than diffuse thalamic involvement and that this involvement may manifest differently in males and females. A better understanding of these sex-dependent patterns could contribute to a more individualized interpretation of epilepsy-related brain alterations and may provide insights into the heterogeneity of the disorder.

The interpretation of thalamic volumetric alterations in epilepsy remains complex. The observed pattern of volumetric increases may be influenced by multiple factors, including disease duration, clinical heterogeneity, imaging protocols, segmentation approaches, and statistical methodology. In the present study, the relatively short mean disease duration may have contributed to the predominance of volumetric increases rather than atrophic changes. Furthermore, the potential effects of antiseizure medications and other clinical variables could not be evaluated because these data were unavailable. Therefore, the observed volumetric enlargements should be interpreted with caution, and their biological significance remains to be clarified.

### 4.8. Conclusions and Limitations

This study examined the structural characteristics and left–right asymmetries of the thalamus in epilepsy patients in detail. Statistically robust alterations after FDR correction and covariate adjustment were observed primarily in VAN, VPLN, MGN, and left MTN in female patients. Trends toward volume differences were also observed in the VLPN and ISN, although these did not survive correction for multiple comparisons or covariate adjustment and therefore warrant cautious interpretation. These changes suggest that epileptic activity may induce long-term adaptive processes in thalamocortical networks. The observation of different patterns in females and males indicates that neuroplastic mechanisms may vary according to sex. The results support the involvement of selected thalamic nuclei in generalized epilepsy; however, the potential functional implications of these structural alterations remain to be clarified. This underscores that the disease cannot be explained solely by cortical structures; subcortical circuits and sex differences must also be considered. The absence of detailed clinical information, including seizure frequency, antiseizure medication exposure, and syndrome-specific characteristics, should be considered when interpreting the present findings, as these factors may influence thalamic morphology. Future studies should pay attention to sex distinctions and continue investigations at the level of individual nuclei to contribute to a more comprehensive understanding of epilepsy.

This study has several limitations. First, although only patients with generalized epilepsy were included to ensure a more homogeneous study population, the findings may not be generalizable to other epilepsy subtypes, such as focal epilepsy. Due to the retrospective design of the study, comprehensive clinical data were not available for all patients. Variables such as seizure frequency, age at onset, antiseizure medication exposure and drug resistance status could therefore not be included in the analyses. As a result, the relationship between thalamic volumetric alterations and specific clinical characteristics of epilepsy remains to be clarified in future prospective investigations.

## Figures and Tables

**Table 1:** Comparison of thalamic nucleus volumes between female epilepsy patients and control group.

Variable	Epilepsy (*n* = 58)	Control (*n* = 58)	*p* (Uncorr.)	Test	pFDR	ANCOVA-pFDR	Effect Size (d or r)	ANCOVA η^2^p
Thalamus Right	4.299 ± 0.646	4.279 ± 0.609	0.870	**	0.902	0.830	d = 0.031 [−0.333, 0.395]	—
Thalamus Left	4.315 ± 0.679	4.303 ± 0.645	0.923	**	0.923	0.830	d = 0.018 [−0.346, 0.382]	—
AVN Left	0.067 ± 0.032	0.060 ± 0.025	0.163	**	0.367	0.311	d = 0.261 [−0.105, 0.626]	—
AVN Right	0.168 ± 0.052	0.154 (0.072–0.300)	0.425	*	0.614	0.682	r = −0.086	—
VAN Right	0.060 ± 0.023	0.048 ± 0.026	0.011 *	**	0.043 *	0.041 *	d = 0.482 [0.113, 0.852]	0.062 (medium)
VAN Left	0.168 ± 0.048	0.150 ± 0.043	0.039 *	**	0.110	0.095	d = 0.387 [0.020, 0.754]	—
VLAN Right	0.056 ± 0.016	0.054 ± 0.016	0.445	**	0.614	0.546	d = 0.142 [−0.222, 0.507]	—
VLAN Left	0.058 ± 0.016	0.056 (0.033–0.093)	0.333	*	0.582	0.830	r = −0.105	—
VLPN Right	0.608 ± 0.128	0.564 ± 0.093	0.035 *	**	0.108	0.063	d = 0.397 [0.029, 0.764]	—
VLPN Left	0.593 ± 0.115	0.566 ± 0.092	0.170	**	0.367	0.273	d = 0.256 [−0.109, 0.622]	—
VPLN Right	0.246 ± 0.066	0.214 ± 0.050	0.005 **	**	0.032 *	0.025 *	d = 0.537 [0.167, 0.908]	0.077 (medium)
VPLN Left	0.252 ± 0.064	0.212 ± 0.053	<0.001 ***	**	0.003 **	0.001 **	d = 0.681 [0.306, 1.055]	0.128 (medium)
PN Right	0.853 ± 0.147	0.871 ± 0.137	0.486	**	0.618	0.682	d = −0.130 [−0.494, 0.234]	—
PN Left	0.872 ± 0.136	0.838 ± 0.150	0.200	**	0.373	0.277	d = 0.240 [−0.126, 0.605]	—
LGN Right	0.031 ± 0.019	0.025 (0.001–0.081)	0.400	*	0.614	0.658	r = −0.091	—
LGN Left	0.033 ± 0.017	0.028 (0.000–0.092)	0.384	*	0.614	0.830	r = −0.094	—
MGN Right	0.040 ± 0.016	0.042 ± 0.013	0.460	**	0.614	0.682	d = −0.138 [−0.502, 0.227]	—
MGN Left	0.045 ± 0.014	0.039 ± 0.011	0.007 **	**	0.032 *	0.041 *	d = 0.512 [0.142, 0.882]	0.066 (medium)
CN Right	0.093 (0.035–0.357)	0.091 ± 0.018	0.579	*	0.675	0.277	r = −0.060	—
CN Left	0.094 (0.056–0.171)	0.092 ± 0.016	0.527	*	0.642	0.396	r = −0.068	—
MN Right	0.438 ± 0.156	0.469 ± 0.095	0.196	**	0.373	0.311	d = −0.241 [−0.607, 0.124]	—
MN Left	0.434 (0.180–0.840)	0.433 ± 0.105	0.845	*	0.902	0.834	r = 0.021	—
HN Right	0.025 ± 0.010	0.021 (0.005–0.054)	0.099	*	0.253	0.311	r = −0.178	—
HN Left	0.022 (0.003–0.055)	0.023 ± 0.010	0.722	*	0.808	0.834	r = 0.039	—
MTN Right	0.012 (0.003–0.156)	0.025 (0.007–0.065)	<0.001 ***	*	<0.001 ***	0.396	r = 0.490	—
MTN Left	0.009 (0.002–0.068)	0.024 (0.000–0.086)	<0.001 ***	*	<0.001 ***	0.001 **	r = 0.475	0.128 (medium)
ISN Right	1.644 (0.000–2.609)	1.798 ± 0.224	0.007 **	*	0.032 *	0.051	r = 0.292	—
ISN Left	1.654 ± 0.285	1.769 ± 0.252	0.024 *	**	0.082	0.063	d = −0.426 [−0.794, −0.058]	—

Values: mean ± SD or median (min–max). *: Mann–Whitney U; **: Independent *t*-test. pFDR: Benjamini–Hochberg FDR correction (28 comparisons). ANCOVA-pFDR: group effect after adjustment for ICV and age, FDR-corrected. Effect size: Cohen’s d [95% CI] for parametric tests; rank-biserial r for non-parametric tests. Green: significant after FDR or ANCOVA-FDR. Yellow: uncorrected *p* < 0.05, did not survive correction. Significance levels: * *p* < 0.05; ** *p* < 0.01; *** *p* < 0.001.

**Table 2:** Right–left hemisphere comparisons of thalamic nucleus volumes in female participants.

Nucleus	Epilepsy Group		Control Group				
	Right	Left	*p* (Raw)	pFDR	*p* (Raw)	pFDR	Effect Size (Epi) [95% CI]
Thalamus	4.299 ± 0.646	4.315 ± 0.679	0.685	0.738	0.613	0.825	d = −0.054 [−0.311, 0.204]
AVN	0.168 ± 0.052	0.067 ± 0.032	<0.001 ***	<0.001 ***	<0.001 ***	<0.001 ***	d = 1.613 [1.223, 2.004]
VAN	0.060 ± 0.023	0.168 ± 0.048	<0.001 ***	<0.001 ***	<0.001 ***	<0.001 ***	d = −2.274 [−2.761, −1.786]
VLAN	0.056 ± 0.016	0.058 ± 0.016	0.328	0.502	0.076	0.207	d = −0.130 [−0.388, 0.129]
VLPN	0.608 ± 0.128	0.593 ± 0.115	0.111	0.311	0.814	0.877	d = 0.212 [−0.048, 0.473]
VPLN	0.246 ± 0.066	0.252 ± 0.064	0.225	0.449	0.648	0.825	d = −0.161 [−0.420, 0.098]
PN	0.853 ± 0.147	0.872 ± 0.136	0.108	0.311	0.040 *	0.140	d = −0.214 [−0.475, 0.046]
LGN	0.031 ± 0.019	0.033 ± 0.017	0.460	0.585	0.455	0.750	d = −0.098 [−0.356, 0.160]
MGN	0.040 ± 0.016	0.045 ± 0.014	0.009 **	0.040 *	0.101	0.207	d = −0.358 [−0.623, −0.092]
CN	0.093 (0.035–0.357)	0.094 (0.056–0.171)	0.554	0.646	0.782	0.877	r = 0.089
MN	0.438 ± 0.156	0.434 (0.180–0.840)	0.149	0.347	<0.001 ***	0.002 **	r = 0.218
HN	0.025 ± 0.010	0.022 (0.003–0.055)	0.257	0.449	0.482	0.750	r = 0.171
MTN	0.012 (0.003–0.156)	0.009 (0.002–0.068)	0.359	0.502	0.960	0.960	r = 0.140
ISN	1.644 (0.000–2.609)	1.654 ± 0.285	0.766	0.766	0.104	0.207	r = 0.045

Values: mean ± SD or median (min–max). pFDR: Benjamini–Hochberg FDR (14 comparisons). Effect size (Epi group): Cohen’s d [95% CI] for parametric; rank-biserial r for non-parametric. *p* (Epi): within epilepsy group right–left comparison; *p* (Ctrl): within control group. Green: survived FDR. Yellow: uncorrected *p* < 0.05, did not survive FDR. When both groups show significant asymmetry: reflects normal lateralization. Significant right–left differences indicate hemispheric volumetric lateralization within the corresponding group. Significance levels: * *p* < 0.05; ** *p* < 0.01; *** *p* < 0.001.

**Table 3:** Comparison of thalamic nucleus volumes between male epilepsy patients and control group.

Variable	Epilepsy (*n* = 58)	Control (*n* = 58)	*p* (Uncorr.)	Test	pFDR	ANCOVA-pFDR	Effect Size (d or r)	ANCOVA η^2^p
Thalamus Right	4.567 ± 0.612	4.526 ± 0.648	0.721	**	0.879	0.957	d = 0.067 [−0.298, 0.431]	—
Thalamus Left	4.580 ± 0.576	4.575 ± 0.774	0.972	**	0.972	0.957	d = 0.006 [−0.358, 0.370]	—
AVN Left	0.081 ± 0.030	0.066 ± 0.033	0.016 *	**	0.146	0.291	d = 0.456 [0.087, 0.825]	—
AVN Right	0.180 ± 0.055	0.168 ± 0.044	0.181	**	0.507	0.565	d = 0.250 [−0.115, 0.615]	—
VAN Right	0.069 ± 0.026	0.063 ± 0.036	0.324	**	0.648	0.591	d = 0.184 [−0.181, 0.549]	—
VAN Left	0.175 ± 0.053	0.155 ± 0.042	0.029 *	**	0.172	0.312	d = 0.410 [0.043, 0.778]	—
VLAN Right	0.054 (0.009–0.122)	0.055 ± 0.017	0.858	*	0.960	0.957	r = −0.020	—
VLAN Left	0.056 ± 0.015	0.057 ± 0.014	0.969	**	0.972	0.957	d = −0.007 [−0.371, 0.357]	—
VLPN Right	0.589 ± 0.112	0.602 ± 0.108	0.506	**	0.798	0.823	d = −0.124 [−0.488, 0.241]	—
VLPN Left	0.599 ± 0.119	0.605 ± 0.094	0.758	**	0.884	0.957	d = −0.057 [−0.421, 0.307]	—
VPLN Right	0.262 (0.039–0.416)	0.246 ± 0.065	0.058	*	0.270	0.565	r = −0.205	—
VPLN Left	0.276 (0.015–0.393)	0.244 ± 0.068	0.031 *	*	0.172	0.450	r = −0.233	—
PN Right	0.948 (0.509–1.627)	0.946 ± 0.142	0.897	*	0.966	0.957	r = −0.014	—
PN Left	0.972 ± 0.140	0.919 ± 0.174	0.071	**	0.285	0.460	d = 0.338 [−0.029, 0.705]	—
LGN Right	0.025 (0.000–0.079)	0.030 ± 0.018	0.542	*	0.798	0.827	r = 0.066	—
LGN Left	0.031 (0.000–0.097)	0.029 (0.002–0.111)	0.556	*	0.798	0.759	r = 0.064	—
MGN Right	0.042 ± 0.017	0.046 ± 0.014	0.181	**	0.507	0.460	d = −0.250 [−0.615, 0.115]	—
MGN Left	0.045 ± 0.016	0.042 ± 0.015	0.231	**	0.587	0.565	d = 0.224 [−0.141, 0.589]	—
CN Right	0.106 ± 0.022	0.098 (0.058–0.628)	0.283	*	0.610	0.690	r = −0.116	—
CN Left	0.102 ± 0.019	0.104 ± 0.022	0.671	**	0.879	0.823	d = −0.079 [−0.443, 0.285]	—
MN Right	0.487 ± 0.126	0.452 (0.032–0.815)	0.112	*	0.393	0.460	r = −0.171	—
MN Left	0.452 ± 0.121	0.439 ± 0.131	0.570	**	0.798	0.825	d = 0.106 [−0.258, 0.470]	—
HN Right	0.025 ± 0.010	0.023 ± 0.011	0.400	**	0.746	0.690	d = 0.157 [−0.207, 0.522]	—
HN Left	0.027 ± 0.011	0.026 ± 0.011	0.722	**	0.879	0.957	d = 0.066 [−0.298, 0.430]	—
MTN Right	0.009 (0.001–0.082)	0.022 (0.003–0.214)	<0.001 ***	*	0.006 **	0.291	r = 0.399	—
MTN Left	0.011 (0.001–0.065)	0.023 ± 0.013	0.002 **	*	0.031 *	0.291	r = 0.330	—
ISN Right	1.754 ± 0.247	1.798 (0.000–2.523)	0.448	*	0.783	0.957	r = 0.082	—
ISN Left	1.749 ± 0.257	1.802 ± 0.269	0.283	**	0.610	0.565	d = −0.200 [−0.565, 0.165]	—

Values: mean ± SD or median (min–max). *: Mann–Whitney U; **: Independent *t*-test. pFDR: Benjamini–Hochberg FDR correction (28 comparisons). ANCOVA-pFDR: group effect after adjustment for ICV and age, FDR-corrected. Effect size: Cohen’s d [95% CI] for parametric tests; rank-biserial r for non-parametric tests. Green: significant after FDR or ANCOVA-FDR. Yellow: uncorrected *p* < 0.05, did not survive correction. Significance levels: * *p* < 0.05; ** *p* < 0.01; *** *p* < 0.001.

**Table 4:** Right–left hemisphere comparisons of thalamic nucleus volumes in male participants.

Nucleus	Epilepsy Group		Control Group				
	Right	Left	*p* (Raw)	pFDR	*p* (Raw)	pFDR	Effect Size (Epi) [95% CI]
Thalamus	4.567 ± 0.612	4.580 ± 0.576	0.812	0.840	0.299	0.503	d = −0.031 [−0.289, 0.226]
AVN	0.180 ± 0.055	0.081 ± 0.030	<0.001 ***	<0.001 ***	<0.001 ***	<0.001 ***	d = 1.886 [1.457, 2.315]
VAN	0.069 ± 0.026	0.175 ± 0.053	<0.001 ***	<0.001 ***	<0.001 ***	<0.001 ***	d = −2.098 [−2.559, −1.638]
VLAN	0.054 (0.009–0.122)	0.056 ± 0.015	0.251	0.359	0.323	0.503	r = 0.175
VLPN	0.589 ± 0.112	0.599 ± 0.119	0.305	0.389	0.830	0.894	d = −0.136 [−0.394, 0.123]
VPLN	0.262 (0.039–0.416)	0.276 (0.015–0.393)	0.257	0.359	0.703	0.820	r = 0.171
PN	0.948 (0.509–1.627)	0.972 ± 0.140	0.022 *	0.078	0.170	0.395	r = 0.345
LGN	0.025 (0.000–0.079)	0.031 (0.000–0.097)	0.195	0.359	0.197	0.395	r = 0.196
MGN	0.042 ± 0.017	0.045 ± 0.016	0.183	0.359	0.040 *	0.144	d = −0.177 [−0.437, 0.082]
CN	0.106 ± 0.022	0.102 ± 0.019	0.163	0.359	0.497	0.696	d = 0.185 [−0.074, 0.445]
MN	0.487 ± 0.126	0.452 ± 0.121	0.003 **	0.015 *	0.045 *	0.144	d = 0.404 [0.137, 0.672]
HN	0.025 ± 0.010	0.027 ± 0.011	0.245	0.359	0.052	0.144	d = −0.154 [−0.413, 0.105]
MTN	0.009 (0.001–0.082)	0.011 (0.001–0.065)	0.801	0.840	0.990	0.990	r = 0.038
ISN	1.754 ± 0.247	1.749 ± 0.257	0.840	0.840	0.670	0.820	d = 0.027 [−0.231, 0.284]

Values: mean ± SD or median (min–max). pFDR: Benjamini–Hochberg FDR (14 comparisons). Effect size (Epi group): Cohen’s d [95% CI] for parametric; rank-biserial r for non-parametric. *p* (Epi): within epilepsy group right–left comparison; *p* (Ctrl): within control group. Green: survived FDR. Yellow: uncorrected *p* < 0.05, did not survive FDR. When both groups show significant asymmetry: reflects normal lateralization. Significant right–left differences indicate hemispheric volumetric lateralization within the corresponding group. Significance levels: * *p* < 0.05; ** *p* < 0.01; *** *p* < 0.001.

## Data Availability

The data presented in this study are available upon request from the corresponding author due to privacy, legal, and ethical restrictions governing the use of routinely collected clinical data.

## Abbreviations

The following abbreviations are used in this manuscript:

AVN	Anterior Ventral Nucleus
CN	Centromedian Nucleus
DBS	Deep Brain Stimulation
EEG	Electroencephalography
HN	Habenular Nucleus
ISN	Intermediate Space Nucleus
LGN	Lateral Geniculate Nucleus
MGN	Medial Geniculate Nucleus
MN	Mediodorsal Nucleus
MRI	Magnetic Resonance Imaging
MTN	Mammillothalamic Tract Nucleus
PN	Pulvinar Nucleus
VAN	Ventral Anterior Nucleus
VLAN	Ventral Lateral Anterior Nucleus
VLPN	Ventral Lateral Posterior Nucleus
VPLN	Ventral Posterolateral Nucleus
VBM	Voxel-Based Morphometry
